# Preparation of Progressive Driving Bilayer Polymer-Dispersed Liquid Crystals Possessing a PDLC-PVA-PDLC Structure

**DOI:** 10.3390/molecules29020508

**Published:** 2024-01-19

**Authors:** Yongle Wu, Yuzhen Zhao, Dong Wang

**Affiliations:** 1 Xi’an Key Laboratory of Advanced Photo-Electronics Materials and Energy Conversion Device, School of Electronic Information, Xijing University, Xi’an 710123, China; wuyongle1999@163.com (Y.W.); zyz19870226@163.com (Y.Z.); 2 Department of Materials Physics and Chemistry, School of Materials Science and Engineering, University of Science and Technology Beijing, Beijing 100083, China

**Keywords:** polymer-dispersed liquid crystals, bilayer, electro–optical properties, PDLC-PVA-PDLC, MoO_2_ nanoparticles

## Abstract

In this paper, the bilayer polymer-dispersed liquid crystals possessing a PDLC-PVA-PDLC structure were prepared by integrating two monolayer PDLCs. The effect of the polymer mesh size on the electro–optical properties of a bilayer PDLC was investigated by comparing the micro-morphology and electro–optical curves under different polymerization conditions. In addition, the impact of doping MoO_2_ nanoparticles with surface modification on the comprehensive performance of the bilayer PDLC was further researched. The contrast ratio of the bilayer PDLC prepared under the optimal conditions was improved by more than 90% and still maintained excellent progressive driving performance. Therefore, the development of a bilayer PDLC with optimal electro-optical properties will significantly enhance the technological prospects for the application of PDLC-based devices in smart windows, displays, and flexible devices.

## 1. Introduction

As a common electro–optical material, polymer-dispersed liquid crystals (PDLC) consist of the polymer substrate and micro or nano-sized liquid crystal (LC) droplets embedded in it [1]. Without voltage being applied, the PDLC exhibits an opaque state due to the random orientation of the LC microdroplets in the polymer substrate [2]. As a large voltage is applied, the PDLC film appears in a transparent state with high transmittance due to the alignment of the LC microdroplets along the direction of the electric field [3]. Inspired by the application of PDLC in the display field, these materials have been rapidly developed in recent years. It has been expanded to other areas beyond displays [4,5] and smart windows [6,7,8,9], such as anti-peep [10], quantum dot films [11,12], and switchable glazing [13,14,15]. In addition, PDLC has been applied in other fields to obtain new composite materials, such as less-energy-hungry buildings [16,17], sensing [18,19], and energy storage [20].

Building on strengths and avoiding weaknesses is a priority in the development of new materials. There are several limitations to all-organic systems, while the optical, thermal, and mechanical stability of the polymer matrix and interaction with the LC can be effectively enhanced by doping nanoparticles [21]. Furthermore, the most common system parameters for display applications can be finely tuned and improved by combining LC, polymers, and nanoparticles to form the three-component system [22]. For example, it was found by Supreet et al. that Cd-free QDs, as dopants, could enable high-performance tunable LCDs and other photonic devices [23]. Moreover, the pathway in which the LC orientation is controlled by nanoparticles has been deeply tapped [24,25]. Hence, doping nanoparticles into PDLC is worthy of further investigation.

In previous work, the electro–optical properties of PDLCs were improved by adjusting the ratio of precursors [26], adding dopants [27,28], and optimizing the polymerization conditions [29]. A faster switching of PDLC was made possible with the addition of functionalized SWCNTs by Shivaraja et al. [30]. Kumar et al. [31] obtained a flexible PDLC with low threshold voltage and high transmittance by introducing a titanium carbide MXene-based transparent conducting electrode. The novel intrinsic high thermal conductivity PDLC was successfully prepared by Li et al. via solution casting and thermal compressing [32]. The optimization of the electro–optical properties of PDLC was achieved through the adjustment of the content of each component, film thickness, and microscopic morphology by Deng et al. [33]. Based on the excellent electronic conductivity [34] and cycling stability [35], the MoO_2_ nanoparticles can be doped into PDLC to improve its electrical performance and stability. In addition, as a transition metal oxide, the MoO_2_ nanoparticles have been widely used in electrode materials [36], battery separators [37], and cocatalysts [38]. For example, MoO_2_-SnO_2_-C nanocomposites fabricated by Feng et al. [39], using a hydrothermal-assisted ball milling method, were expected to be an anode material for lithium-ion batteries due to the realization of long-term cycling stability. The incorporation of MoO_2_ nanoparticles into PDLC not only the composite films with more excellent properties can be obtained, but also the application potential of MoO_2_ can be further exploited.

In recent years, multilayer structural materials have been rapidly developed and used in a variety of applications, such as multilayer polymers [40], dielectric elastomers [41], and coatings [42]. Huang et al. [43] used a 4D printing strategy to prepare multilayer polymers, which exhibited tunable mechanical properties and shape memory behavior. Scalable fabrication of multilayer dielectric elastomer actuators has been achieved with batch-sprayed and stamp-transferred electrodes by Cohen et al. [44]. Compared with the monolayer coatings, the multilayer coatings prepared by Feng et al. [45] using the plasma spraying technology possess excellent ablation resistance, and their applications are more promising.

**Figure 1:**
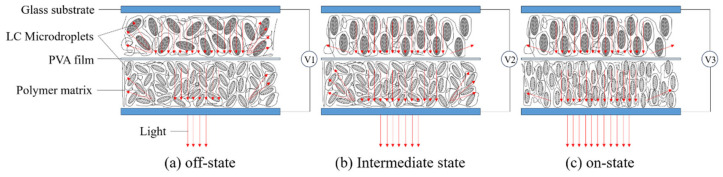
Schematic diagram of the structure of double-layer PDLC and its driving principle.

Inspired by previous studies, the concept of multilayer structures is introduced into the field of PDLCs in this paper to solve the current problems. By integrating two PDLC layers with a PVA layer, the bilayer PDLC was obtained, as shown in Figure 1. Compared with the conventional monolayer PDLC, this bilayer PDLC can not only enter the on-state and off-state but also realize the controllable transmittance by adjusting the voltage under low-voltage conditions. Films made of PVA have excellent strength and are not dissolved in the LC/monomer mixture, which can act as a spacer between the two PDLC layers. In addition, the stabilized PDLC-PVA-PDLC structure allows the bilayer PDLC to perform optimally in anti-aging tests, which will enable its application potential to be further stimulated.

## 2. Results and Discussions

### 2.1. Effect of Crosslinker Content on the Property of Monolayer PDLC

**Figure 2:**
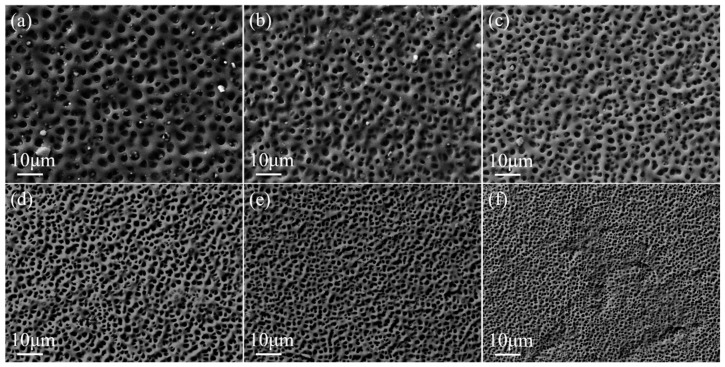
SEM images of PDLC cells of (**a**) 0% PEGDA200 (**b**) 1% PEGDA200 (**c**) 2% PEGDA200 (**d**) 3% PEGDA200 (**e**) 4% PEGDA200 (**f**) 5% PEGDA200 which are under magnification of 3000×.

The crosslinker content has an effect on the degree of crosslinking of the polymer molecular chains, which in turn affects the polymer microscopic morphology. The SEM images of the polymer mesh with PEGDA200 content are shown in Figure 2. The higher content of PEGDA200 provides more crosslinking points in the molecular chain, resulting in a smaller polymer mesh size. As the PEGDA200 content increased from 0 to 5 wt%, the LC droplet size gradually decreased from 3.0 μm to 1.0 μm, as can be observed in Figure 2. In addition, the LC microdroplet size was uniform for each sample, which was essential for the stability of PDLC properties.

**Figure 3:**
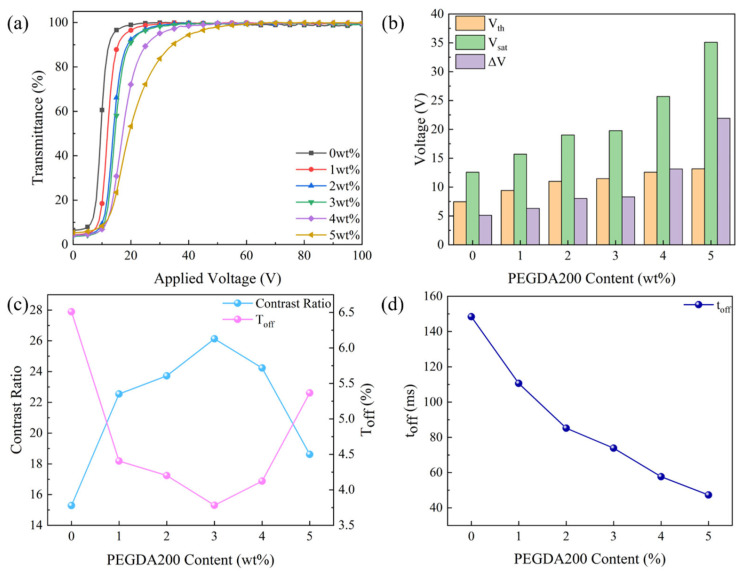
The effect of crosslinker (PEGDA200) content on electro–optical properties: (**a**) voltage–transmittance curve; (**b**) threshold voltage (V_th_), saturation voltage (V_sat_), and ΔV; (**c**) contrast ratio (CR) and off-state transmittance (T_off_); and (**d**) response time (t_off_).

The smaller the polymer mesh size, the greater the anchoring force on LC microdroplets, leading to changes in the electro–optical properties of PDLC. The variation of the electro–optical properties of monolayer PDLC with PEGDA200 content is illustrated in Figure 3. The transmittance–voltage curves were shifted to the right with the increase of PEGDA200 content, and the values of V_th_ and V_sat_ were increased from 7.5 V and 12.6 V to 13.2 V and 35.1 V, respectively, according to Figure 3a,b. Since the increase in the value of V_sat_ is greater than V_th_, ΔV increased from 5.1 V to 21.9 V. The polymer microscopic morphology has an effect on the refractive index matching between the polymer and the LC microdroplets, which then impacts the T_off_ and CR of the PDLC. As depicted in Figure 3c, the value of T_off_ decreased from 6.5% to 3.8% when the content of PEGDA200 was increased from 0 to 3 wt%. As the PEGDA200 content increased further to 5 wt%, the value of T_off_ increased to 5.4%. The refractive index match between the polymer and LC was optimal after polymerization as the PEGDA200 was augmented from 0 to 3 wt%, at which the CR reached a maximum and the T_off_ a minimum. However, with the PEGDA200 continuing to be raised to 5 wt%, the refractive index match between the polymer and the LC after polymerization was weakened, resulting in a decrease in CR and an increase in T_off_. Due to the fact that CR is inversely proportional to T_off_, its value increased from 15.3 to 26.1 and then decreased to 18.6. The smaller the polymer mesh size, the greater the anchoring force on the LC microdroplets, and the shorter the time required to transition from the ordered to the disordered state. Thus, as the content of PEGDA200 was increased from 0 to 5 wt%, the value of t_off_ decreased from 148.4 ms to 47.3 ms, as indicated in Figure 3d.

After comprehensively considering the relationship between the transmittance–voltage curves, V_sat_, V_th_, CR, and t_off_, the samples corresponding to PEGDA200 contents of 1% and 3% were used as the upper and lower layers for the preparation of bilayer PDLC.

### 2.2. Effect of UV Light Intensity on the Property of Bilayer PDLC

**Figure 4:**
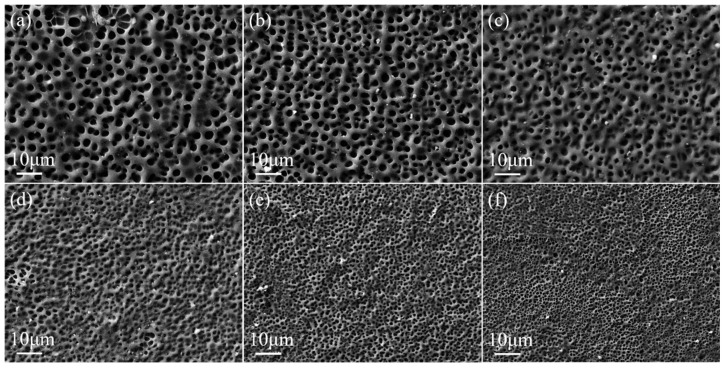
SEM images of upper layer PDLC cells of (**a**) 1.5 mW/cm^2^, (**b**) 3.0 mW/cm^2^, (**c**) 4.5 mW/cm^2^, (**d**) 6.0 mW/cm^2^, (**e**) 7.5 mW/cm^2^, and (**f**) 9.0 mW/cm^2^, which are under a magnification of 3000×.

**Figure 5:**
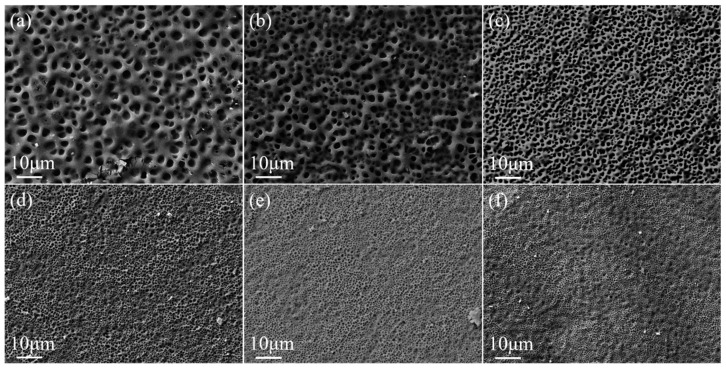
SEM images of lower layer PDLC cells of (**a**) 1.5 mW/cm^2^, (**b**) 3.0 mW/cm^2^, (**c**) 4.5 mW/cm^2^, (**d**) 6.0 mW/cm^2^, (**e**) 7.5 mW/cm^2^, and (**f**) 9.0 mW/cm^2^, which are under a magnification of 3000×.

**Figure 6:**
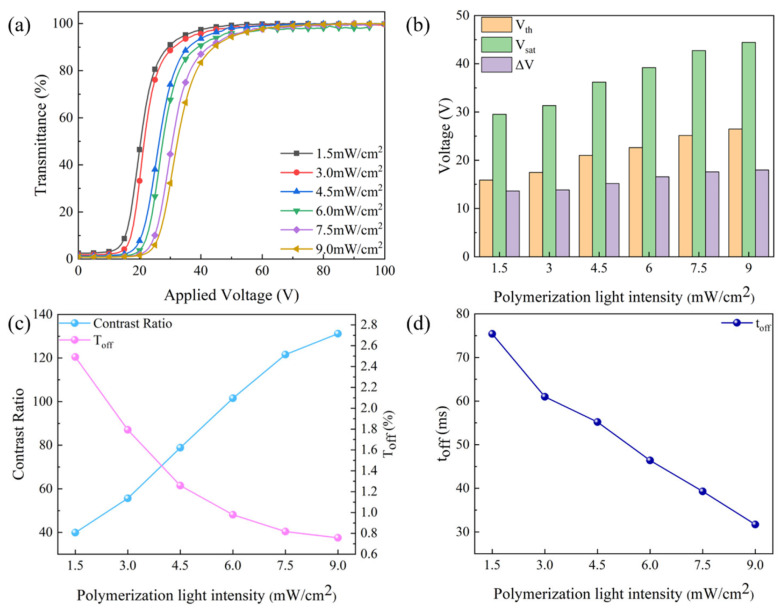
The effect of polymerization light intensity on electro–optical properties: (**a**) voltage–transmittance curve; (**b**) threshold voltage (V_th_), saturation voltage (V_sat_), and ΔV; (**c**) contrast ratio (CR) and off-state transmittance (T_off_); and (**d**) response time (t_off_).

**Figure 7:**
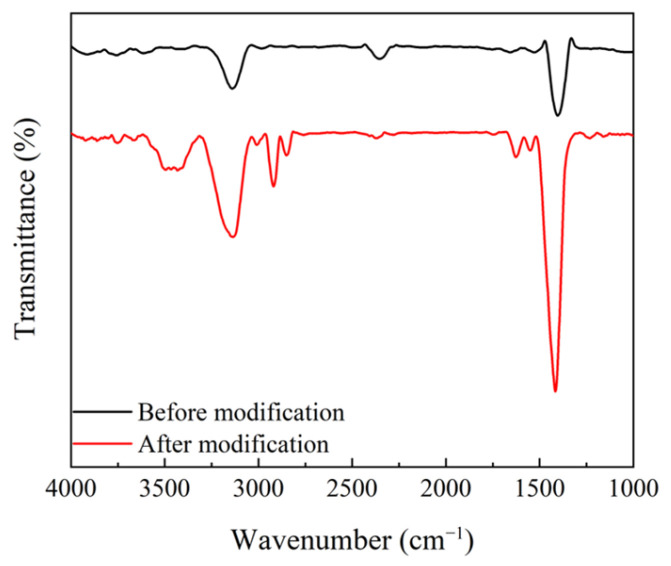
Fourier transform infrared (FTIR) spectra of MoO_2_ nanoparticles before and after modification.

UV light intensity affects the rate of polymerization of monomers, which in turn affects the mesh size. The higher the UV light intensity, the faster the rate of polymerization of the monomer, which resulted in a smaller mesh size. The polymer microscopic morphology of the upper and lower PDLC layers is shown in Figure 4 and Figure 5. The LC microdroplet size was maximum at a UV intensity of 1.5 mW/cm^2^ based on Figure 6 and Figure 7. The upper and lower LC microdroplet sizes were 3.3 μm and 3.0 μm, respectively. The LC microdroplets became smaller as the light intensity gradually increased. The sizes of the upper and lower LC droplets were reduced to 0.8 μm and 0.5 μm when the light intensity was 9 mW/cm^2^, respectively. Besides, the LC microdroplets were able to maintain uniformity during the growth of light intensity.

The electro–optical properties of PDLC were affected by the UV light intensity, as shown in Figure 6. As the UV intensity increased, the transmittance–voltage curves shifted to the right and the values of V_th_ and V_sat_ grew from 15.9 V and 29.5 V to 26.5 V and 44.4 V, which are shown in Figure 6a,b. It was due to the fact that the smaller the polymer mesh, the greater the anchoring force on the LC microdroplets, and the greater the voltage required for the LC microdroplets to change from the disordered state to the ordered state. Since the increase in the value of V_sat_ is larger than V_th_, ∆V rose from 13.6 V to 18.0 V. In addition, the UV light intensity affects the refractive index matching between the polymer and the LC microdroplets, leading to changes in T_off_ and CR. T_off_ decreased from 2.5% to 0.8%, since CR varied opposite to T_off_, it increased from 40.0 to 131.2, as demonstrated in Figure 6c. The larger the anchoring force on the LC microdroplet, the easier it is to transfer from the ordered state to the disordered state, so the smaller the t_off_ is. Therefore, the t_off_ decreased from 75.4 ms to 31.7 ms as the light intensity increased, as depicted in Figure 6d.

By comparing the differences between the performances of different samples, it was found that the comprehensive performance of the sample was relatively excellent when the UV intensity was 6 mW/cm^2^.

### 2.3. MoO_2_ Nanoparticles Doped Bilayer PDLC

#### 2.3.1. Modification of MoO_2_ Nanoparticles

In order to inhibit the agglomeration between MoO_2_ nanoparticles and enable them to be uniformly dispersed in the precursor of LC/monomer, they were surface-modified by using oleic acid. The Fourier transform infrared spectra of MoO_2_ nanoparticles before and after modification are plotted in Figure 7. The spectra of the modified MoO_2_ nanoparticles exhibited distinct characteristic peaks at 1700 cm^−1^ and 2900 cm^−1^ as compared to the premodified ones. Among them, the characteristic peak at 1700 cm^−1^ is the carbonyl stretching vibration peak and at 2900 cm^−1^ is the methylene stretching vibration peak. Oleic acid molecules were successfully modified on the surface of MoO_2_ nanoparticles according to the above analysis.

#### 2.3.2. Effect of MoO_2_ Nanoparticles Content on the Property of Bilayer PDLC

**Figure 8:**
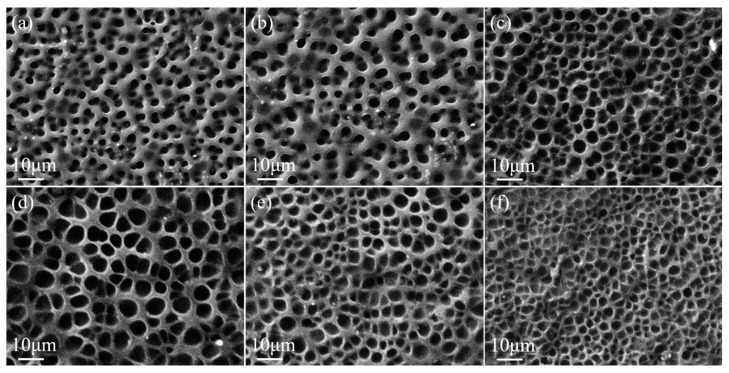
SEM images of upper layer PDLC cells of (**a**) 0.2 wt%, (**b**) 0.4 wt%, (**c**) 0.6 wt%, (**d**) 0.8 wt%, (**e**) 1.0 wt%, and (**f**) 1.2 wt% MoO_2_ nanospheres doped, which are under a magnification of 3000×.

**Figure 9:**
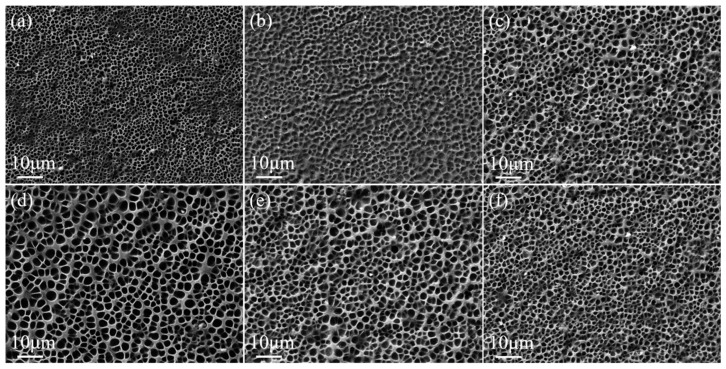
SEM images of lower layer PDLC cells of (**a**) 0.2 wt%, (**b**) 0.4 wt%, (**c**) 0.6 wt%, (**d**) 0.8 wt%, (**e**) 1.0 wt%, and (**f**) 1.2 wt% MoO_2_ nanospheres doped, which are under a magnification of 3000×.

The MoO_2_ nanoparticle content had an effect on the polymer microscopic morphology, as shown in Figure 8 and Figure 9, which show the variation of the polymer network in the upper and lower PDLC layers with nanoparticle content. When the content of MoO_2_ nanoparticles was low, they combined with the LC microdroplets, which led to an increase in the size of the polymer mesh. As the content of MoO_2_ nanoparticles increased, they bound to both the LC microdroplets as well as the polymer network, causing the polymer mesh to diminish. The LC microdroplet size kept increasing as the MoO_2_ nanoparticle content was added from 0.2 wt% to 0.8 wt% from Figure 8 and Figure 9. The LC microdroplets in the upper and lower layers reached 6.0 μm and 4.0 μm when the content of MoO_2_ nanoparticles was 0.8 wt%, respectively. However, the LC microdroplet size diminished as the MoO_2_ nanoparticle content was further enlarged to 1.2 wt%. The LC microdroplet sizes of the upper and lower layers were minimized to 4.0 μm and 2.0 μm. The LC microdroplet size changed while remaining homogeneous.

**Figure 10:**
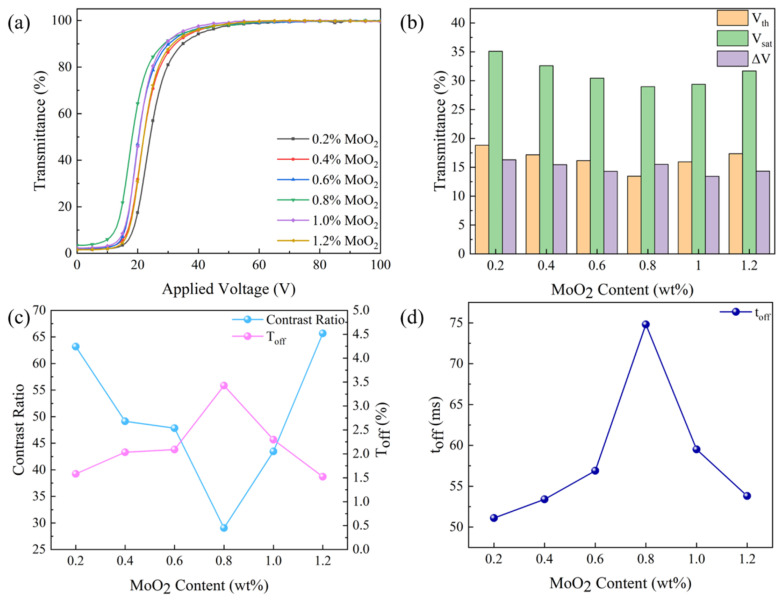
The effect of MoO_2_ nanoparticles content on electro–optical properties: (**a**) voltage–transmittance curve; (**b**) threshold voltage (V_th_), saturation voltage (V_sat_), and ΔV; (**c**) contrast ratio (CR) and off-state transmittance (T_off_); and (**d**) response time (t_off_).

MoO_2_ nanoparticles affect the electro–optical properties of PDLC by influencing the micro-morphology of the polymer network. The variation of electro–optical properties of PDLC with the content of MoO_2_ nanoparticles is displayed in Figure 10. The transmittance–voltage curves of the bilayer PDLC are shifted to the left and then to the right, and accordingly, their V_th_ and V_sat_ decrease and then increase, as illustrated in Figure 10a,b. When the content of MoO_2_ nanoparticles was increased from 0 to 0.8 wt%, the transmittance–voltage curves shifted leftward, and the V_th_ and V_sat_ decreased from 18.8 V and 35.1 V to 13.5 V and 29.0 V, due to the fact that the bilayer PDLC was easier to drive. As the content of MoO_2_ nanoparticles further increased to 1.2 wt%, the bilayer PDLC became difficult to drive, resulting in a rightward shift of the transmittance–voltage curves, and the V_th_ and V_sat_ increased to 17.4 V and 31.7 V. Since the difference between V_sat_ and V_th_ varied less, the ΔV was relatively stable and remained around 15 V. The variation of T_off_ and CR with the content of MoO_2_ nanoparticles is characterized in Figure 10c. As the nanoparticle content increased from 0 to 0.8 wt%, T_off_ rose from 1.6% to 3.4%, and CR reduced from 63.2 to 29.1. With the further increase in nanoparticle content to 1.2 wt%, T_off_ lessened to 1.5%, and CR improved to 65.7. The reason for these variations is that different contents of MoO_2_ nanoparticles have different effects on the refractive indices of the LC microdroplets and polymers, which results in differences in the refractive index matching between them. Besides, the t_off_ is also affected by the content of MoO_2_ nanoparticles, and the variation curve between them is described in Figure 10d. The process of increasing the content of MoO_2_ nanoparticles, the t_off_ first increases from 51.1 ms to 74.8 ms and then decreases to 53.8 ms. It is caused by the fact that the nanoparticles first increase the size of the LC microdroplets, which reduces the anchoring force exerted on the LC microdroplets and makes them difficult to recover from the ordered state to the disordered state. Subsequently, the nanoparticles reduce the size of the polymer mesh, which leads to an increase in the anchoring force exerted on the LC microdroplets and a decrease in the time required for the transition to the disordered state.

**Figure 11:**
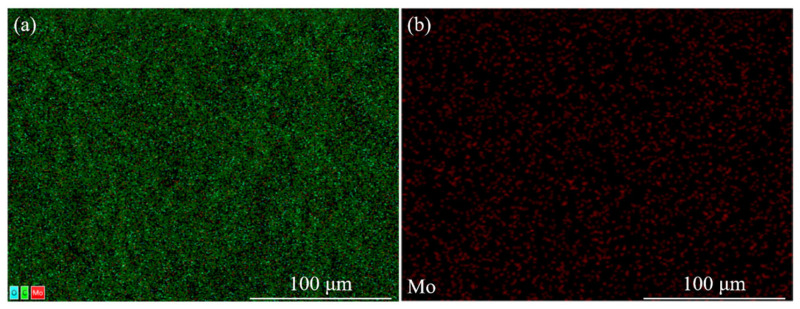
EDS mapping of c3 sample: (**a**) the distribution of all elements and (**b**) the distribution of Mo elements.

By analyzing the electro–optical properties of the samples with different nanoparticle contents, it was found that the comprehensive performance of the bilayer PDLC was optimal when the MoO_2_ nanoparticle content was 0.6 wt%. In addition, by analyzing the EDS mapping of sample c3 as Figure 11, it can be seen that MoO_2_ nanoparticles were uniformly distributed in the polymer network, which is a prerequisite for the stable performance of the bilayer PDLC.

### 2.4. Progressive Driving Performance Testing of Bilayer PDLC

**Figure 12:**
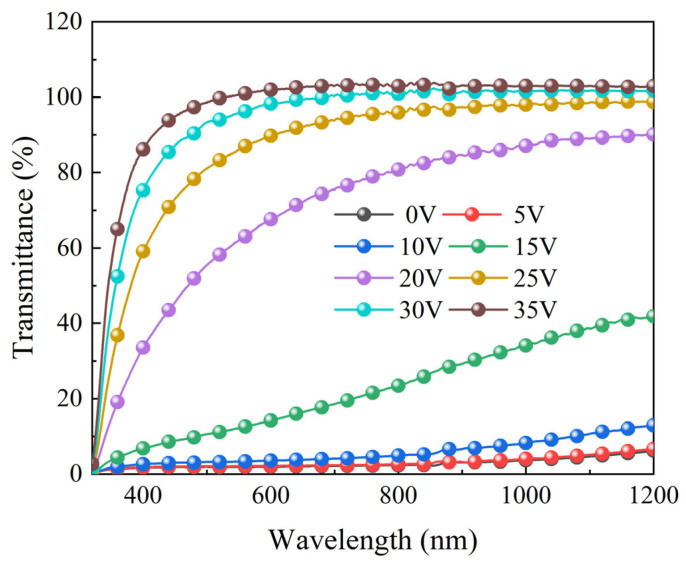
The transmittance–wavelength curves of the optimum sample at different voltages.

In order to verify the progressive driving performance of the bilayer PDLC, the transmittance of different wavelength’s light was explored at different voltages first, as demonstrated in Figure 12. The transmittance of the bilayer PDLC for different wavelength’s light grew gradually with the increase of the applied voltage. When a smaller voltage was applied, the orientation of a portion of the LC microdroplets changed from the disordered state to parallel to the direction of the electric field and, thus, the bilayer PDLC presented a smaller transmittance. As the applied voltage continued to increase, more and more LC microdroplets were oriented parallel to the direction of the electric field, resulting in a further growth of the transmittance of the bilayer PDLC. When the voltage was increased to 30 V, almost all of the LC microdroplets were positioned parallel to the electric field, resulting in a nearly stable transmittance of the bilayer PDLC.

**Figure 13:**
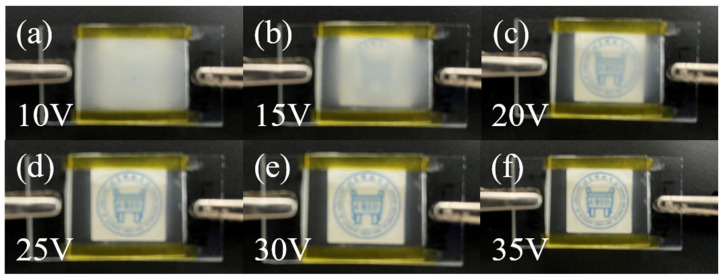
The physical plots of the transmittance variation of the optimum sample at different voltages.

To visualize the progressive driving performance of the bilayer PDLC, it was characterized using physical diagrams, depicted in Figure 13. The transmittance of the bilayer PDLC is a gradual process with growing applied voltage.

### 2.5. Aging Resistance Testing of Bilayer PDLC

**Figure 14:**
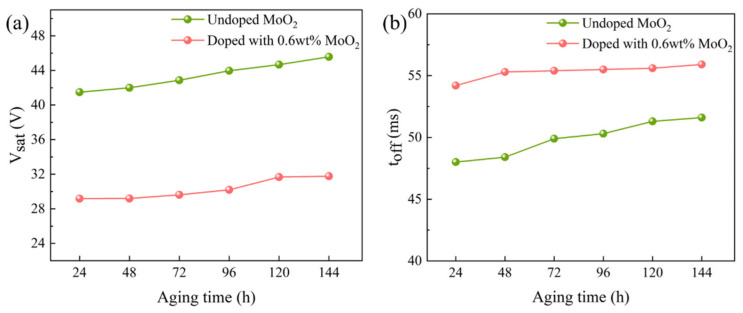
The aging resistance curves of samples with undoped and doped MoO_2_ nanoparticles: (**a**) saturation voltage (V_sat_)-aging time, and (**b**) response time (t_off_)-aging time.

The prepared bilayer PDLC samples, before and after MoO_2_ nanoparticle modification, were treated with high temperature (50 °C) and strong ultraviolet light (830 mW/m^2^), and their properties were tested to examine their aging resistance. The variation of V_sat_ and t_off_ for the two samples are shown in Figure 14. Compared to the undoped sample, the MoO_2_ nanoparticle doping resulted in a better performance of the sample in the aging resistance test. The performance of the sample doped with MoO_2_ nanoparticles was more stable for the same testing time.

### 2.6. Application of Bilayer PDLC in Flexible Smart Light Control Film

**Figure 15:**
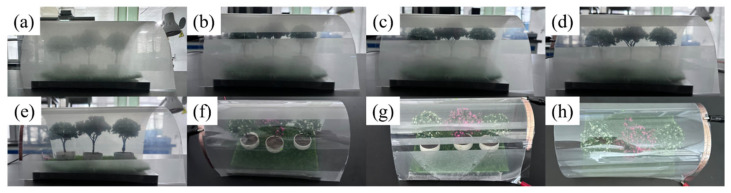
The physical diagram of bilayer PDLC applied in flexible smart light control film: (**a**) the off-state, (**b**) the smallest range on-state, (**c**) the smaller range on-state, (**d**) the wide range on-state, (**e**) the main view of wider range on-state, (**f**) the top view of wider range on-state, (**g**) the selective localized on-state, (**h**) the all on-state.

For smart light control under flexible conditions, the bilayer PDLC sample was prepared using etched PET conductive film. The smart light control can be achieved by applying voltages at different positions of the bilayer PDLC, as shown in Figure 15. In addition, this process was done under bending conditions. Light control of different areas was realized by adjusting the position of the applied voltage according to demand. The feature can be practiced in both continuous and discontinuous regions.

## 3. Experimental

### 3.1. Materials

The nematic liquid crystals SLC1717 (*n*_o_ = 1.519, *n*_e_ = 1.720, Δ*n* = 0.201, T_c_ = 92 °C) used in this experiment were purchased from Shijiazhuang Chengzhi Yonghua Display Materials Co. (Shijiazhuang, China) UV65N (*n* = 1.520) as a mixture of UV-polymerizable monomers was purchased from Kuer Industries (Shanghai) Co. (Shanghai, China). Bifunctional poly(ethylene glycol) diacrylate (PEGDA, Mr = 200, *n* = 1.461) was purchased as a crosslinker from Shanghai Aladdin Biochemical Technology Co. (Shanghai, China). The free radical photoinitiator, 2,2-dimethopxy-2-phenylacetophenone (IRG651), was purchased from Anhui Zesheng Technology Co. (Anqing, China).

**Figure 16:**
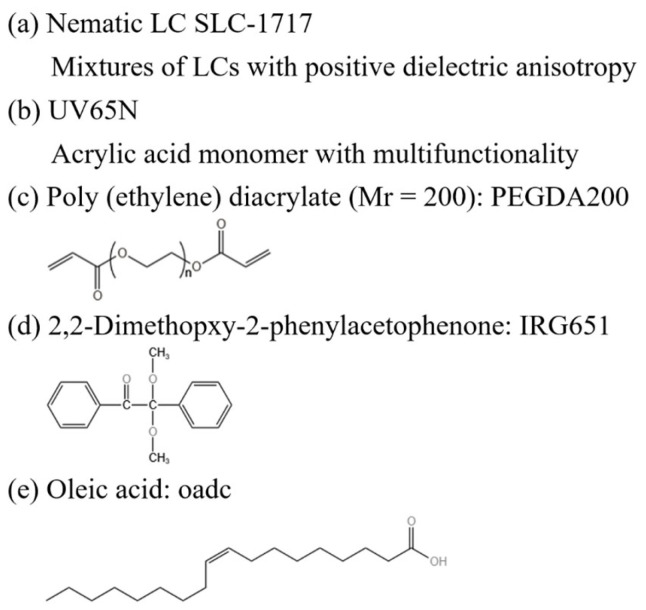
The specific chemical structure of the materials used.

MoO_2_ nanoparticles (diameter 50~80 nm) were purchased from Tianjin Heowns Biochemical Technology Co. (Tianjin, China). The modifier oleic acid was purchased from Sinopharm Chemical Reagent Co. (Shanghai, China). The specific chemical structures of the materials used in this paper are given in Figure 16. In order to inhibit the agglomeration of MoO_2_ nanoparticles and to make them uniformly dispersed in the LC/monomer mixture, they were treated with surface modification using oleic acid.

The specific process of surface treatment of MoO_2_ nanoparticles was as follows. The MoO_2_ nanoparticles (10 mmol) were dispersed in tetrahydrofuran (20 mL) and mechanically stirred (300–600 rpm) for 30 min at 65 °C to mix them uniformly. Under continuous heating conditions, the oleic acid (5 mL)/tetrahydrofuran (10 mL) mixture was added to the above MoO_2_ solution, and stirring was continued for 30 min. Oleic acid (2 mL) was added to the above MoO_2_ solution per 5 min interval, and this operation was repeated 5 times. The mixture was mechanically stirred (300~600 rpm) for 2 h at 85 °C. The above heating process needs to be operated under condensing conditions to avoid volatilization of the tetrahydrofuran. The black mixture was cooled to room temperature after removing the heat source. The precipitate obtained after centrifugation (5000 rpm, 5 min) separation the above mixture was washed repeatedly for 5 times using anhydrous ethanol to obtain the primary modified MoO_2_ nanoparticles. The above MoO_2_ nanoparticles were dissolved in a mixture of oleic acid (5 mL)/hexane (20 mL) and mechanically stirred (800~1000 rpm) for 2 h. The precipitate obtained after centrifugation (5000 rpm, 10 min) and separation of the above mixture was repeatedly washed using anhydrous ethanol for 5 times, and dried to obtain the secondary modified MoO_2_ nanoparticles.

### 3.2. Sample Preparation

The PVA film is insoluble in LC and has high strength, which is the key to the preparation of bilayer PDLC. The specific preparation process is as follows.

(1) Preparation of precursors. The LC/monomer/initiator mixtures with different ratios were shaken for 3 min, stirred for 5 min and sonicated for 15 min to make them into homogeneous phases. Specific ratios are shown in Table 1.
(2) Preparation of monolayer PDLC. The LC cells were obtained by fixing two single-sided conductive glass substrates spaced apart using an 8-micron-thick polyimide film as a spacer. By capillary action, the mixtures a1~a6 in Table 1 were infused into the LC cells, and the monolayer PDLCs were obtained after polymerization. The polymerization time, light intensity, and temperature for this group of samples were 5 min, 4.5 mW/cm^2^, and 35 °C, respectively.

(3) Preparation of PVA film. A layer of aqueous PVA solution (0.05 wt%) was dropped on the surface-treated glass substrate using surface tension. The glass substrate with PVA film attached on the surface was obtained by standing under heating at 75 °C for 3 h.
(4) Preparation of bilayer PDLC. Samples a2 and a4 were used as two monolayer PDLCs for the preparation of bilayer PDLC. The glass substrate with PVA film attached to the surface was spaced from the unilaterally conductive glass substrate using an 8-micron-thick polyimide film and fixed to obtain the LC cell. The precursor of a2 was infused into the LC cell using capillary action, and the PDLC-PVA composite film was obtained after curing. The surface-treated glass substrate was peeled off to obtain a glass substrate with a PDLC-PVA composite film attached to the surface. The glass substrate with PDLC-PVA composite film attached to the surface was spaced from the unilateral conductive glass substrate using an 8-micron-thick polyimide film to obtain a new LC cell. The precursor of a4 was infused into the liquid crystal box using capillary action, and a bilayer PDLC possessing a PDLC-PVA-PDLC structure was obtained after curing.

Among them, the variables of Group B and C were the UV light intensity and MoO_2_ nanoparticle content, respectively, as shown in Table 2.

**Figure 17:**
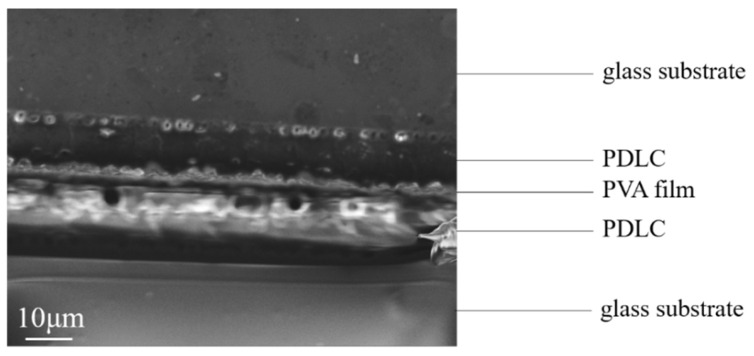
The SEM image of the cross-section of the bilayer PDLC possessing PDLC-PVA-PDLC structure.

The SEM image of the obtained bilayer PDLC cross-section is shown in Figure 17. The bilayer PDLC prepared in this way possessed a stable PDLC-PVA-PDLC structure, and the interface between PVA and PDLC was clearer.

### 3.3. Characterization

To verify the attachment of oleic acid to the surface of the MoO_2_ nanoparticles, the chemical composition was analyzed using a Fourier transform infrared spectrometer (FTIR, INVENIO S).

The electro–optical properties, one of the most crucial properties, can often be representative of the application potential of PDLC. Electro–optical curves, response time curves, and contrast ratio were measured by using a liquid crystal comprehensive parameter tester (LCT-5016C, Beijing LCD Engineering Research and Development Center) in this experiment. In addition, other key parameters such as threshold voltage (V_th_), saturation voltage (V_sat_), off-state transmittance (T_off_), and off-state response time (t_off_) are also obtained through electro–optical properties testing. Herein, the V_th_ and V_sat_ were obtained when the transmittance of the bilayer PDLC reached 10% and 90% of the maximum value, respectively. The ratio between the transmittance in the on-state and in the off-state was termed the contrast ratio (CR).

The morphology of the polymer matrix was observed using a scanning electron microscope (SEM, S-4800), and the samples were treated as follows prior to observation. The prepared PDLC samples were soaked in cyclohexane for 15 d at room temperature to remove all LC molecules. The cyclohexane was replaced every 3 d during the immersion process to ensure that the LC molecules could be adequately removed. At the end of the immersion, the samples were dried at 60 °C for 1 h to remove residual cyclohexane from the surface. The dried samples were gold-sprayed under vacuum for SEM observation.

The transmittance of the samples at different wavelengths was tested using a Lambda 950 UV/Vis/NIR spectrophotometer (Perkin-Elmer, Waltham, MA, USA) at room temperature, and the transmittance–wavelength curves of the bilayer PDLC at different voltages were obtained.

## 4. Conclusions

In brief, the bilayer PDLC possessing a PDLC-PVA-PDLC structure was prepared by introducing the multilayer concept into PDLC. Compared with the conventional monolayer PDLC, the bilayer PDLC can not only enter the on-state and off-state, but also the intermediate state with controllable transmittance. It can be found that the doping of bilayer PDLC using surface-modified MoO_2_ nanoparticles can further improve its comprehensive performance by analyzing its microscopic morphology and electro–optical curves. When the polymerization conditions as well as the nanoparticle content, were optimal, the CR of the obtained samples increased by more than 90% and still maintained excellent progressive driving properties according to the experimental results. In addition, the application fields of bilayer PDLC have been greatly widened due to the excellent aging resistance and progressive driving performance, which enables it to satisfy the requirements for smart displays. A new process for the preparation of PDLC was offered in this work that provided novel ideas for subsequent developments in the field of liquid crystals.

## Figures and Tables

**Table 1:** The composition of Group A ^a^.

Sample	Weight Percentage (wt%)		
	SLC1717	UV65N	PEGDA200
Group A			
a1	50	50	0
a2	50	49	1
a3	50	48	2
a4	50	47	3
a5	50	46	4
a6	50	45	5

^a^ The weight of the photoinitiator IRG651 is 0.5% of the total weight.

**Table 2:** The composition of Groups B and C.

Sample	UV Light Intensity (mW/cm^2^)	MoO_2_ Nanoparticles Content (wt%)
Group B		
b1	1.5	0
b2	3.0	0
b3	4.5	0
b4	6.0	0
b5	7.5	0
b6	9.0	0
Group C		
c1	6.0	0.2
c2	6.0	0.4
c3	6.0	0.6
c4	6.0	0.8
c5	6.0	1.0
c6	6.0	1.2

## Data Availability

Data are contained within the article.
